# Supplementary material for: COVID-19 risk score as a public health tool to guide targeted testing: A demonstration study in Qatar
Source: PLoS One. 2022 Jul 19;17(7):e0271324. doi: 10.1371/journal.pone.0271324 (PMC9295939; doi:10.1371/journal.pone.0271324)
Supplement: S1 Table — (DOCX) [file pone.0271324.s001.docx]

**Table S1.** Results of multivariable logistic regression analysis using only 25% of the sample to derive the original Qatar COVID-19 risk score.

|  | **“Original” Qatar COVID-19 risk score** | | |
| --- | --- | --- | --- |
| **Characteristics** | **Coefficient** | **aOR (95% CI)** | **Score points** |
| Sex |  |  |  |
| Male | 0.000 | 1.00 | 0 |
| Female | -0.301 | 0.74 (0.64-0.86) | -3 |
| Age (years) |  |  |  |
| <10 | 0.000 | 1.00 | 0 |
| 10-19 | 0.052 | 1.05 (0.91-2.51) | 1 |
| 20-29 | 0.029 | 1.023 (0.86-1.79) | 0 |
| 30-39 | 0.093 | 1.13 (0.63-1.90) | 1 |
| 40-49 | 0.096 | 1.19 (0.99-1.76) | 1 |
| 50-59 | 0.298 | 1.36 (1.08-2.96) | 3 |
| 60-69 | 0.581 | 1.78 (1.35-3.39) | 6 |
| 70-79 | 1.131 | 3.10 (1.33-7.21) | 11 |
| 80+ | -0.030 | 0.99 (0.15-7.98) | 0 |
| Nationality |  |  |  |
| Other^*^ | 0.000 | 1.00 | 0 |
| Bangladeshi | 1.384 | 3.60 (2.78-4.51) | 14 |
| Nepalese | 1.201 | 3.22 (2.43-4.20) | 12 |
| Indian | 0.874 | 2.58 (1.97-2.90) | 9 |
| Pakistani | 0.861 | 2.30 (1.60-2.95) | 9 |
| Kenyan | 0.659 | 1.96 (0.95-3.74) | 7 |
| Egyptian | 0.470 | 1.64 (1.15-2.21) | 5 |
| Sri Lankan | 0.077 | 1.08 (0.63-1.53) | 1 |
| Sudanese | 0.234 | 1.25 (0.85-2.05) | 2 |
| Filipino | -0.405 | 0.69 (0.49-1.01) | -4 |
| Qatari | -0.498 | 0.70 (0.60-0.79) | -5 |

β, beta coefficient; aOR, adjusted odds ratio; CI, confidence interval.

^*^These include 148 other nationalities residing in Qatar.
