# Supplementary material for: COVID-19 risk score as a public health tool to guide targeted testing: A demonstration study in Qatar
Source: PLoS One. 2022 Jul 19;17(7):e0271324. doi: 10.1371/journal.pone.0271324 (PMC9295939; doi:10.1371/journal.pone.0271324)
Supplement: S2 Table — (DOCX) [file pone.0271324.s002.docx]

**Table S2.** Results of multivariable random-effect logistic regression analysis (with random level at location of PCR testing) used to derive the original Qatar COVID-19 risk score.

|  | **“Original” Qatar COVID-19 risk score** | | |
| --- | --- | --- | --- |
| **Characteristics** | **Coefficient** | **aOR (95% CI)** | **Score points** |
| Sex |  |  |  |
| Male | 0.000 | 1.00 | 0 |
| Female | -0.353 | 0.68 (0.64-0.72) | -4 |
| Age (years) |  |  |  |
| <10 | 0.000 | 1.00 | 0 |
| 10-19 | 0.026 | 0.97 (0.79-1.20) | 0 |
| 20-29 | -0.114 | 0.89 (0.76-1.05) | -1 |
| 30-39 | -0.067 | 0.93 (0.79-1.10) | -1 |
| 40-49 | 0.125 | 1.13 (0.96-1.34) | 1 |
| 50-59 | 0.301 | 1.35 (1.14-1.60) | 3 |
| 60-69 | 0.469 | 1.60 (1.32-1.93) | 5 |
| 70-79 | 0.766 | 2.15 (1.66-2.78) | 8 |
| 80+ | -0.313 | 0.73 (0.44-1.21) | -3 |
| Nationality |  |  |  |
| Other^*^ | 0.000 | 1.00 | 0 |
| Bangladeshi | 1.240 | 3.46 (3.17-3.77) | 12 |
| Nepalese | 1.189 | 3.28 (3.00-3.59) | 12 |
| Indian | 0.811 | 2.25 (2.08-2.44) | 8 |
| Pakistani | 0.894 | 2.44 (2.19-2.73) | 9 |
| Kenyan | 0.687 | 1.99 (1.61-2.46) | 7 |
| Egyptian | 0.355 | 1.43 (1.24-1.64) | 4 |
| Sri Lankan | 0.019 | 0.98 (0.83-1.16) | 0 |
| Sudanese | 0.197 | 1.22 (1.02-1.45) | 2 |
| Filipino | -0.104 | 0.90 (0.79-1.03) | -1 |
| Qatari | -0.090 | 0.91 (0.84-1.00) | -1 |

β, beta coefficient; aOR, adjusted odds ratio; CI, confidence interval.

^*^These include 148 other nationalities residing in Qatar.
